# Nosocomial Infections During Extracorporeal Membrane Oxygenation in Pediatric Patients: A Multicenter Retrospective Study

**DOI:** 10.3389/fped.2022.873577

**Published:** 2022-06-13

**Authors:** Chunle Wang, Shuanglei Li, Feng Wang, Jinfu Yang, Wei Yan, Xue Gao, Zhiqiang Wen, Yaoyao Xiong

**Affiliations:** ^1^Extracorporeal Life Support Center of Cardiovascular Surgery, The Second Xiangya Hospital of Central South University, Central South University, Changsha, China; ^2^Cardiovascular Surgery Department, The Sixth Medical Center of People's Liberation Army of China (PLA) General Hospital, Beijing, China; ^3^Department of Pediatric ICU, Affiliated Children's Hospital of Zhengzhou University, Zhengzhou, China; ^4^Cardiovascular Surgery, The Second Xiangya Hospital of Central South University, Central South University, Changsha, China

**Keywords:** extracorporeal membrane oxygenation, children, nosocomial infection, risk factors, ECMO

## Abstract

**Objective:**

Extracorporeal membrane oxygenation (ECMO) is increasingly used in critically ill patients with respiratory and/or cardiac failure. This study aimed to investigate the epidemiology and risk factors of nosocomial infection (NI) in pediatric patients who underwent ECMO for respiratory and/or circulatory failure.

**Methods:**

Medical records for patients that were administered underwent ECMO support at Xiangya Second Hospital of Central South University, The Sixth Medical Center of PLA General Hospital, and Children's Hospital Affiliation of Zhengzhou University, from September 2012 to December 2019 were retrospectively reviewed. Clinical data of the patients who developed NI were collected and analyzed. Univariate and multivariate logistic regressions were performed to identify the independent predictive factors of NI during ECMO.

**Results:**

A total of 54 first episodes of NI were identified in the 190 patients on ECMO, including 32 cases of respiratory tract infections, 20 cases of bloodstream infections, and 2 cases of surgical site wound infections. Gram-negative pathogens were the dominant pathogens isolated, accounting for 92.6% of the NI. The incidence of ECMO-related NI was 47.6 cases per 1,000 ECMO days. In the univariate logistic regression, ECMO mode, ECMO duration, ICU duration, and peritoneal dialysis were associated with the development of NI in patients with ECMO support. However, in the multivariate analysis, only ECMO duration (OR = 2.46, 95%CI: 1.10, 5.51; *P* = 0.029), ICU duration (OR = 1.35, 95%CI: 1.05, 1.59; *P* = 0.017) and peritoneal dialysis (OR = 2.69, 95%CI: 1.08, 5.73; *P* = 0.031) were the independent predictive factors for NI during ECMO support.

**Conclusion:**

This study identified the significant correlation between ECMO-related NI and ECMO duration, ICU duration, and peritoneal dialysis. Appropriate preventive measures are needed for hospitals to reduce the incidence of ECMO in pediatric patients.

## Introduction

Extracorporeal membrane oxygenation (ECMO) is an extracorporeal life support (ECLS) strategy for critically ill children with reversible cardiac failure and/or pulmonary failure (1). ECMO support is required by roughly 1% of patients undergoing cardiac surgery as a means of supporting the life circulation (2, 3). During ECMO, blood is drained, diverted to an artificial lung, and then, returned to the patients, which provides respiratory and hemodynamic support (4). However, the cannulation of large blood vessels breaches the barrier protection of patients and provides an easy entry point for pathogens. ECMO is associated with numerous potentially life-threatening complications, such as bleeding, infection, thrombosis, and stroke (5–7). ECMO secondary infections may affect about 31% of patients (8), which not only increase patients' suffering but also result in death (9). Although some of the complications, such as thrombosis and hemorrhage, have been reduced with the improved technology, nosocomial infections (NIs) present multiple challenges to effective diagnosis and management.

It has only been recently that the NI during ECMO has received attention (6). Since the influenza A (H1N1) pandemic in 2009, especially the new coronavirus pneumonia (COVID-19) global pandemic in 2019, ECMO has been adopted by many hospitals around the world (10–13). A great number of studies have focused on this issue. However, these studies reported different rates and risk factors of NI during ECMO (14–16). In adult patients, NIs are commonly seen in those who have predisposing factors such as patients' comorbidities, and other life-support procedures (e.g., invasive mechanical ventilation [IMV], renal replacement therapies [RRT]). However, for pediatric populations, the study on NIs acquired during ECMO remains scarce. Thus, we performed this study to investigate the epidemiology and risk factors of NIs in the children population who underwent ECMO for respiratory and/or circulatory failure.

## Materials and Methods

### Study Design and Setting

This study was conducted in compliance with Strengthening the Reporting of Observational Studies in Epidemiology (STORBE) Statement: guidelines for reporting observational studies (17) (Supplementary Material). This retrospective study was performed at Xiangya Second Hospital of Central South University, The Sixth Medical Center of PLA General Hospital, and Children's Hospital Affiliation of Zhengzhou University, using data collected from organizations' ECMO database from September 2012 to December 2019. The inclusion criteria were: (1) patients treated with ECMO using a circuit containing a membrane oxygenator and pump, (2) patients who removed a catheter from ECMO treatment within the past 48 h, and (3) patients under 18 years old. Exclusion criteria were: (1) patients who collected data during previous data collection, (2) patients with ECMO extubation > 48 h, and (3) patients with extracorporeal support excluding membrane oxygenators and/or pumps. The study was approved by the Institutional Ethical Committee of the Second Xiangya Hospital of Central South University in China (No. 2020-011).

The clinical data were collected: gender, age, weight, diagnosis at admission (acute respiratory distress syndrome, congenital heart disease, septic shock, pulmonary hypertension, myocarditis, and other diseases), mode of ECMO support (venovenous or venoarterial), mode of cannulation (peripheral or central), renal replacement therapy (CRRT or peritoneal dialysis), transferred from the peripheral hospital (yes or no), extracorporeal cardiopulmonary resuscitation (ECPR: yes or no), ECMO duration (days), hospital duration (days), invasive mechanical ventilation (IMV) duration (days) and intensive care unit (ICU) duration (days).

The criteria of diagnosis at admission were as follows: 1. acute respiratory distress syndrome: a. Identify the acute dyspnea within 1 week; b. Chest X-ray plain film/chest CT showed double lung infiltration shadow; c. Respiratory failure cannot be completely explained by cardiac failure and fluid overload; d. Hypoxemia; 2. congenital heart disease: a. The history of pregnancy: the first 3 months of the pregnant woman had a viral infection, radiation exposure, history of medication, history of diabetes, malnutrition, and environmental and genetic factors; b. Common symptoms: shortness of breath, cyanosis, rapid heart rate (up to 180 beats/min), pause during feeding due to dyspnea, and asthma; c. Development of infant: Infants with congenital heart disease often suffer from malnutrition, a thin body, and growth retardation; and d. Ultrasonography: quantitative measurement of each chamber size and blood vessel in the heart to diagnose the anatomical abnormalities and severity of congenital heart disease. 3. Septic shock: a. under ultrasound or imaging examination, there will be obvious infection focus and systemic infection; b. the decline of blood perfusion leads to organ dysfunction; c. blood pressure drops below 80 / 50 mmHg; d. blood samples were taken to detect the growth of related pathogenic bacteria in blood culture. Pulmonary hypertension: under resting status, the mean pulmonary artery pressure measured by the right cardiac catheter is > or equal to 25 mmHg. Myocarditis: a. Cardiac insufficiency, cardiogenic shock, or cardio cerebral syndrome. b. Cardiac enlargement (X-ray, echocardiography). c. Related ECG changes. d. C-MB was elevated, or cardiac cadherin was positive.

Among the patients, all of them received blood routine examinations and patients with respiratory symptoms received X-ray tests. The diagnosis of NI referred to the diagnostic criteria of the Centers for Disease Control and Prevention/National Nosocomial Infections Surveillance system (18). The ECMO-related NI was defined as a case that had confirmed organisms from one or more blood, respiratory, surgical site wound, or urinary cultures during the initiation of ECMO to 24 h after ECMO weaning (19). In case of suspected infection, the responsible doctor will require routine microbiological examinations (tracheal aspirates, blood cultures, and urine) without E. coli culture. The case was examined by an infectious disease expert and an anesthesiologist to determine if there was a real clinical co-infection. The sites selected for blood sampling were swabbed with 70% alcohol. Five to 10 milliliters were collected in bactec bottles, transported to the laboratory, and placed in bactec 9,120 instruments. The positive bottles were dyed in Gram and subcultured and tested for sensitivity by the microbiologist. Endotracheal aspirates were obtained by sucking the endotracheal tube or tracheostomy tubes using a sterile suction catheter and the tip cut with a sterile surgical blade, placed in a sterile container, and sent to the laboratory. The purulent part of the vacuum cleaner was used to inoculate MacConkey blood dishes, chocolate, and agar by the lab technician. Chocolate and blood plates were incubated in carbon dioxide at 35–37°C and MacConkey in ambient air for 24 h. Positive cultures that had isolated and sensitive cultures were identified. Medium flow urine or from a sampling port to an internal catheter using an aseptic technique was collected in a sterile container. Samples were used to inoculate MacConkey blood agar and agar, which was incubated at 35–37°C for 18–24 h. Positive cultures were stained with Gram and subcultured and tested for sensitivity. Pus swabs or wounds from ulcers and septic wounds were removed. In laboratory blood, MacConkey and chocolate agar were inoculated, incubated, and treated as mentioned above. All positive microbial cultures obtained from the beginning of ECMO support until 48 h after decannulation were independently evaluated based on available clinical, laboratory, and radiographic data. Accordingly, the following NIs were diagnosed: ventilator-associated pneumonia (VAP), bloodstream infection (BSI, including catheter-related bloodstream infections), and surgical site wound infections. Surgical site wound infections were diagnosed when all the following were present: (1) local erythema and purulent drainage; and (2) cultures of the purulent drainage positive for microorganisms other than common skin contaminants. Only the first NI episode was included in the analysis. The patients were divided into the NI group and non-NI group according to the NI condition.

The diagnosis criteria of VAP were as follows: First, onset after using a ventilator for 48 h; Second, compared with chest X-ray before mechanical ventilation, there was infiltration shadow in the lung or showed new inflammatory lesions; Third, signs of pulmonary consolidation and/or wet rales could be heard in pulmonary auscultation, and one of the following conditions was met: a. blood cells > 10.0^*^109 / L or < 4^*^109 / L, with or without nuclear metastasis; b. Fever, body temperature > 37.5°C, a large number of purulent secretions in the respiratory tract; and c. New pathogenic bacteria were isolated from bronchial secretions after onset. The diagnosis criteria of BSI were: First, a recognized pathogen from cultured blood, and the organism cultured was not related to an infection at another site. Organisms considered common skin contaminants were not included in the definition of a recognized pathogen. Second, a fever (>38°C), chills, or hypotension if the patient is > 1 year of age; b. fever (>38°C core) hypothermia (< 36°C core), apnea, or bradycardia if the patient is 1 year of age.

Cannulation of ECMO was performed in a sterile fashion with complex iodine painting. The selection of cannulation model, size, and approach site was determined by the surgeon according to the patient's body weight, height, and vessel size. The management protocol for ECMO follows the Extracorporeal Life Support Organization (ELSO) guidelines.

### Statistical Analysis

Statistical analysis was performed using SPSS 18.0 for Windows (SPSS Chicago, IL, USA). The characteristics of patients in NI and non-NI groups were compared. Continuous variables were expressed as mean ± SD, and a non-parametric test (Mann-Whitney U-test) was used to test the statistical significance. Categorical variables were analyzed using Fisher's exact test. Univariate and multivariate analyses were performed to identify the independent predictive factors of NI during ECMO. Candidate factors for the multivariable analysis were chosen based on previous findings of univariate analysis and possible biological association (20–23). The odds ratio (OR) was calculated with corresponding 95% confidence intervals (CIs). A receiver operating characteristic (ROC) curve was used to determine the area under the curve (AUC) and the cut-off values of ECMO duration for predicting NI. A two-tailed *P-*value **<** 0.05 was considered statistically significant.

## Results

### Patient Characteristics, and Course and Outcome of ECMO

**Figure 1:**
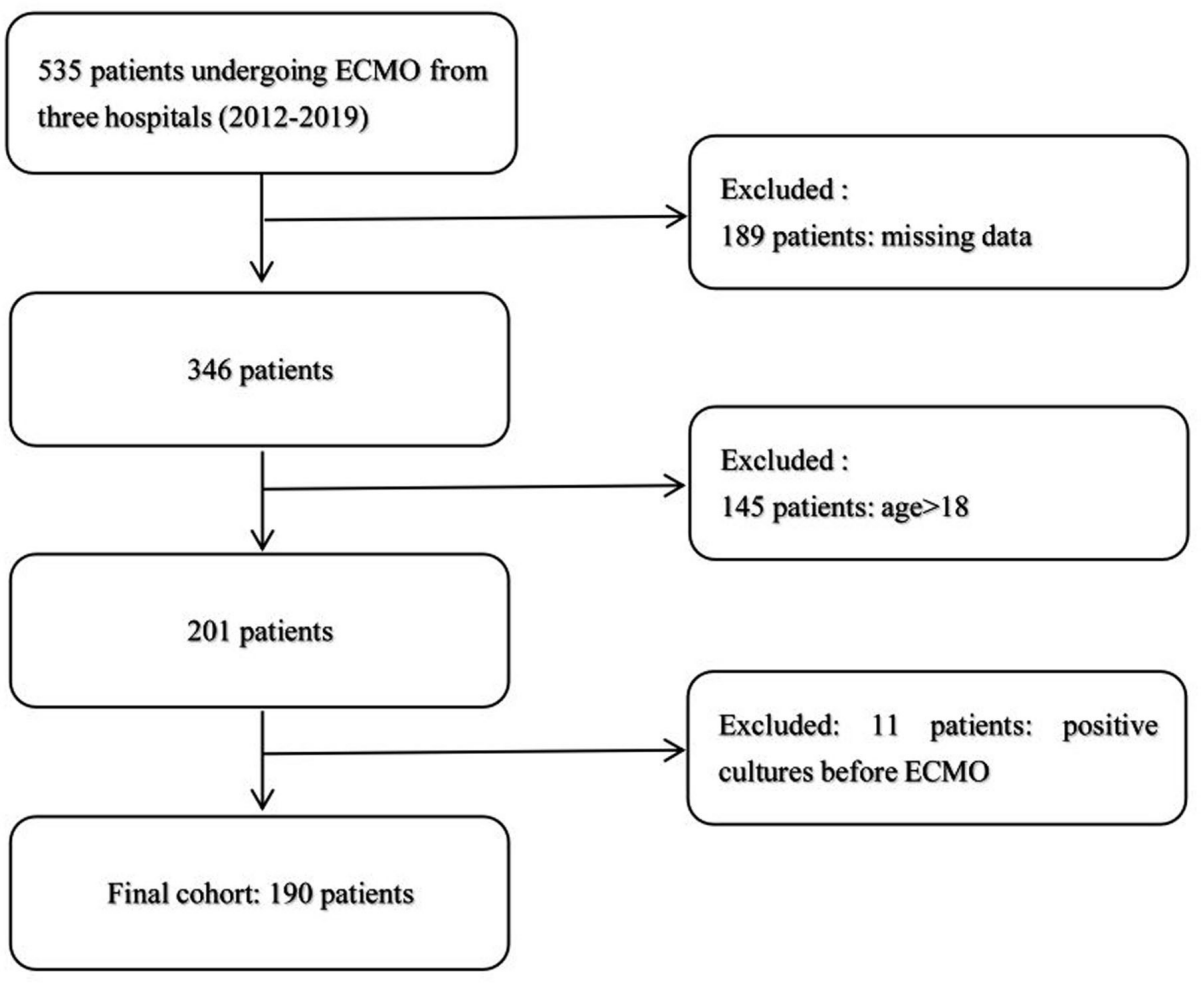
Study flowchart.

According to the inclusion and exclusion criteria, we excluded 345 patients, and 190 patients were selected, in which no one had positive cultures before ECMO (Figure 1). The characteristics of 136 patients without NI and 54 patients with NI are shown in Table 1. During the study, 78 neonates (0–28 days) and 112 pediatric patients (28 days−17 years) received ECMO support. The incidence of ECMO-related NI was 47.6 cases per 1,000 ECMO days. About 65% of nosocomial infections occurred within 12 days and 20% within 6 days. The mean delay of onset was 14.5 ± 10.8 days.

**Table 1:** The characteristics of patients with and without nosocomial infections during ECMO.

**Factors**	**All patients**	**Non-infection**	**Infection**	***x^2^*/Z**	***P*-value**
No. of patients	190	136	54		
**Age**				18.539	0.000
N (0–28days)	78 (41.1%)	69 (50.7%)	9 (16.7%)		
P (28 days−17 years)	112 (58.9%)	67 (49.3%)	45 (83.3%)		
**Gender**				1.866	0.225
Male	130 (68.4%)	97 (71.3%)	33 (61.1%)		
Female	60 (31.6%)	39 (28.7%)	21 (38.9%)		
Weight(Kg)		4(8.72)	10.75(13.50)	−4.016	0.000
Transferred from peripheral hospital				0.699	0.451
Yes	45 (23.7%)	30 (22.1%)	15 (27.8%)		
No	145 (76.3%)	106 (77.9%)	39 (72.2%)		
**Diagnosis at admission**				9.028	0.139
Acute respiratory distress syndrome	75 (39.5%)	52 (38.2%)	23 (42.6%)		
Congenital heart disease	65 (34.2%)	43 (31.6%)	22 (40.7%)		
Septic shock	7 (3.7%)	5(3.7%)	2 (3.7%)		
Pulmonary hypertension	15 (7.9%)	14 (10.3%)	1 (1.9%)		
Myocarditis	23 (12.1%)	17 (12.5%)	6 (11.1%)		
Others	5 (2.6%)	5 (3.7%)	0		
**Antibiotics use**				0.223	0.018
BLA	38	33	5		
PA	13	6	7		
BLA + PA	133	94	39		
Others	6	3	3		
**ECMO support mode**				5.399	0.02
V-V ECMO	11 (5.8%)	4 (2.9%)	7 (13.0%)		
V-A ECMO	179 (94.2%)	132 (97.1%)	47 (87.0%)		
**Cannulation**				1.619	0.209
Peripheral cannulation	139 (73.2%)	103 (75.7%)	36 (66.7%)		
Central cannulation	51 (26.8%)	33 (24.3%)	18 (33.3%)		
**CRRT**				0.895	0.344
Yes	11 (5.8%)	6 (4.4%)	5 (9.3%)		
NO	179 (94.2%)	130 (95.6%)	49 (90.7%)		
**Peritoneal dialysis**				1.348	0.319
YES	39 (20.5%)	25 (18.4%)	14 (28.6%)		
NO	151 (79.5%)	111 (81.6%)	40 (71.4%)		
**ECPR**				0.779	0.444
YES	20 (10.5%)	16 (11.8%)	4 (7.4%)		
NO	170 (89.5%)	120 (88.2%)	50 (92.6%)		
ECMO duration (d)	4.61 ± 4.12	4 ± 4.06	5.26± 6.70	−3.260	0.001
IMV duration (d)	11 ± 12	9 ± 7.93	17 ± 15.25	−4.424	0.001
ICU duration (d)	15 ± 17	13 ± 14.75	20 ± 16	−3.670	0.000
Hospital duration (d)	24 ± 26.25	20 ± 24.75	31 ± 29.25	−3.466	0.001

*ECMO, Extracorporeal membrane oxygenation; BLA, Beta-lactam antibiotics; PA, Polypeptide antibiotics; CRRT, continuous renal replacement therapy; ECPR, extracorporeal cardiopulmonary resuscitation; IMV, invasive mechanical ventilation; ICU, intensive care unit*.

Before diagnosed as NI, there were 89 patients with infections received antibiotics among 190 patients included. All the patients with NI received antibiotics, and the standard therapy is as follows: the treatment of participants included in this time must ensure that most Gram-negative bacteria are covered, and the combination treatment of anti-pseudomonal penicillin plus an aminoglycoside, or an antipseudomonal cephalosporin, plus an aminoglycoside is used. If there are Gram-positive bacteria, we add glycopeptides.

There were no significant differences in sex, diagnosis at admission, transferred from the peripheral hospital, antibiotics use, cannulation, ECPR, CRRT, and peritoneal dialysis between the two groups. In the non-NI group, 69 (50.7%) neonates received ECMO support, which was > that in the NI group (n = 9, 16.7%). Acute respiratory distress syndrome and congenital heart disease were the principal diagnoses at the time of admission, which made up 39.5 and 34.2%, respectively. The ECMO support modes differed significantly between the two groups (*P* **=** 0**.**02), with 132 (97.1%) and 47 (87.0%) patients, using V-A ECMO in non-NI and NI groups, respectively. CRRT was used in only 5.8% of patients, and peritoneal dialysis was in 20.5% of patients.

The ECMO duration, IMV duration, ICU duration, and hospital duration were the most significant differences between the two groups. The average ECMO support duration was significantly shorter in the non-NI group (4 ± 4.06 days) than that in the NI group (5.26 ± 6.70 days). The average IMV duration in the non-NI group was 9 ± 7.93 days and 17 ± 15.25 days in the NI group. The average duration of ICU stay was significantly lower in patients without NI (13 ± 14.75 days) than in patients with NI (20 ± 16 days). Similarly, the hospital duration was significantly shorter in patients without NI (20 ± 24.75 days) than in patients with NI (31 ± 29.25 days).

One hundred and fifteen patients underwent ECMO support weaning with an ECMO survival rate of 60.52%. The Non-NI group survival rate (59.56%) was lower than that of NI group (62.96%), however, this difference was not significant (*P* **=** 0**.**665). The overall survival rate was 48.42%, with 48.53% in the non-NI group, and 48.15% in the NI group (Table 1).

### Microorganisms of First Episode Nosocomial Infection

The microorganism distributions of the first episode NI are presented in Table 2. There are 75 episodes in 54 patients, of which 21 patients had another episode of NI. The NI types included 32 respiratory tract infections, 36 BSI, and 7 surgical site wound infections. A total of 90 strains of pathogens were isolated, among which gram-negative pathogens were the dominant (75 strains, 83.3%), mainly including *Acinetobacter baumannii* (31 strains), *Klebsiella pneumonia* (17 strains), *Pseudomonas aeruginosa* (8 strains), *Stenotrophomonas maltophilia* (6 strains), *Pseudomonas paucimobilis (*2 strains), *Burkholderia cepacian* (3 strains), *Burkholderia pickettii (*1 strain), followed by fungal (5 strains, 6.6%), and gram-positive pathogens (2 strains, 2.6%).

**Table 2:** Distribution of the Microorganisms of First Nosocomial Infection.

**Gram staining**	**Microorganism**	**Respiratory tract infection (*n*, %)**	**Blood stream infection (*n*, %)**	**Surgical site wound infection (*n*, %)**	**Overall**
		32 (42.67)	36 (48)	7 (9.33)	75 (100)
G-negative bacteria		31 (41.33)	30 (40)	7 (9.33)	68 (90.7)
	*Acinetobacter baumannii*	18 (24)	13 (17.33)		31 (41.3)
	*Klebsiella pneumoniae*	7 (9.33)	7 (9.33)	3 (4)	17 (22.6)
	*Pseudomonas aeruginosa*		6 (8)	2 (2.67)	8 (10.6)
	*Stenotrophomonas maltophilia*	3 (4)	2 (2.67)	1 (1.33)	6 (8)
	*Pseudomonas paucimobilis*		2 (2.67)		2 (2.6)
	*Burkholderia cepacian*	2 (2.67)		1 (1.33)	3 (4)
	*Burkholderia pickettii*	1 (1.33)			1 (1.3)
G-positive bacterial			2 (2.67)		2 (2.6)
	*Staphylococcus epidermidis*		2 (2.67)		2 (2.6)
Fungal		1 (1.33)	4 (5.33)		5 (6.6)
	*Candida spp*		2 (2.67)		2 (2.6)
	*Candida parapsilosis*	1 (1.33)	2 (2.67)		3 (4)
	*Candida lusitaniae*				

### Established ROC Curve

**Figure 2:**
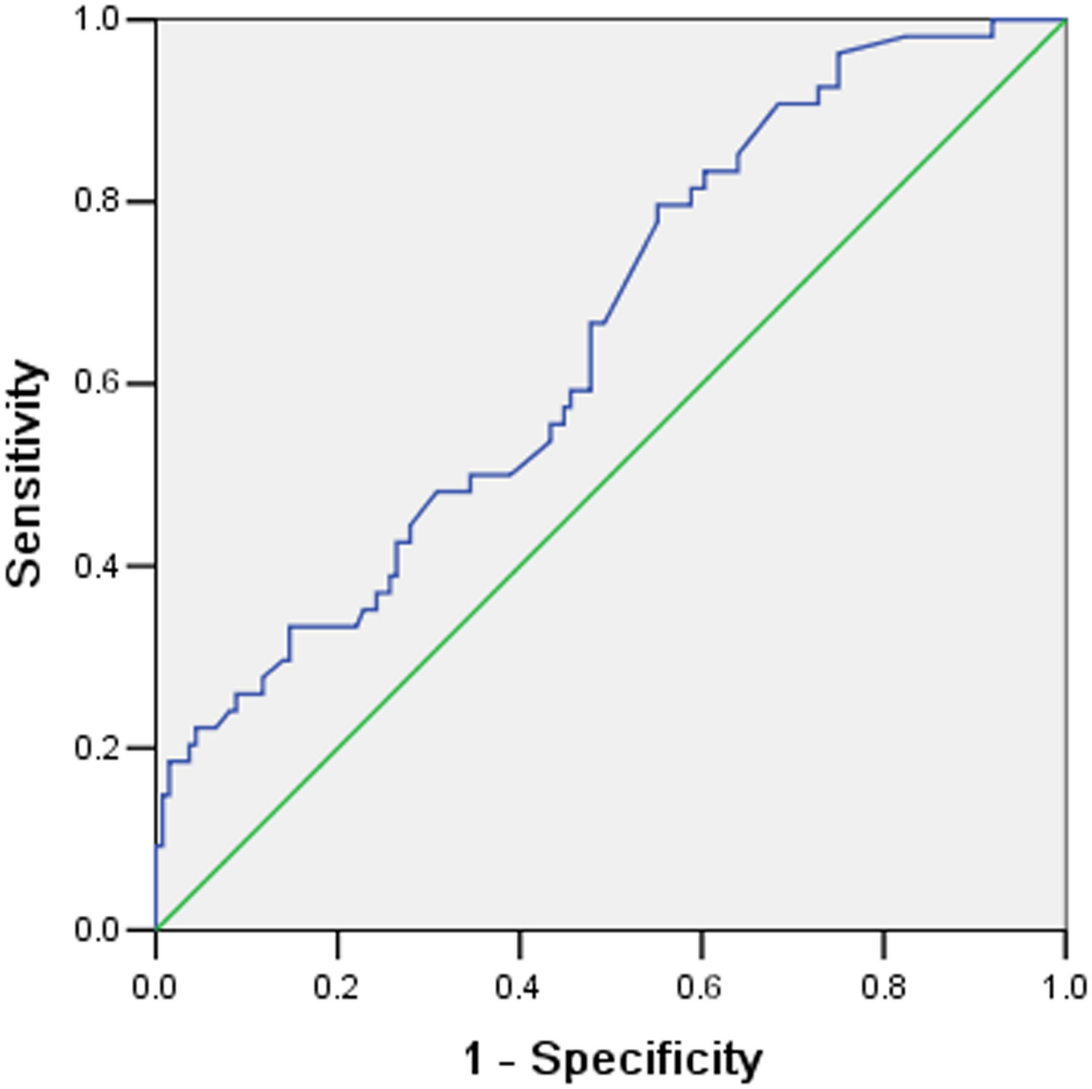
The Receiver operating characteristic (ROC) curves for predicting nosocomial Infection according to extracorporeal membrane oxygenation (ECMO) duration.

The ROC curve was performed to assess the ability of ECMO duration to predict NI. The discriminating ability and cut-off of the ECMO duration were as follows: AUC.652(95%CI**:**0**.5**68, 0**.7**35) (Figure 2), and a cut-off value of 3.94 days (Table 3). A significant difference in the discriminative power was observed (*P* = 0**.**001).

**Table 3:** The specific results of ROC Curve.

**Variables**	**Cut-off value**	**AUC (95%CI)**	**Youden's index**	***P***
ECMO duration (d)	3.94	0.652 (0.568~0.735)	0.245	0.001

### Multivariate Analysis of Risk Factors for Nosocomial Infection

It can be seen from Table 1 that the age, weight, ECMO support mode, ECMO duration, IMV duration, ICU duration, and hospital duration differed significantly between the two groups. In the univariate logistic regression, ECMO mode, ECMO duration, ICU duration, and peritoneal dialysis were associated with the development of NI in patients with ECMO support (Table 4). However, in the multivariate analysis, only ECMO duration (OR = 2.46, 95%CI: 1.10, 5.51; *P* **=** 0**.**029), ICU duration (OR = 1.35, 95%CI: 1.05, 1.59; *P* **=** 0**.**017), and peritoneal dialysis (OR = 2.69, 95%CI: 1.08, 5.73; *P* **=** 0**.**031) were the independent predictive factors for NI during ECMO support. Moreover, subgroup logistic analyses based on infection types were conducted. In respiratory tract infection, univariate analysis showed that ECMO support mode (OR = 1.68, 95%CI: 1.41–4.14), ECMO duration (OR = 1.32, 95%CI: 1.16–1.56), and IMV duration (OR = 1.04, 95%CI: 1–1.08) had a relationship with NI. Multivariate analysis showed only ECMO duration had an association with NI (OR = 1.38, 95%CI: 1.17-1.73). Univariate analysis for bloodstream infection showed weight (OR = 1.05, 95%CI: 1.01–1.09), peritoneal dialysis (OR = 1.33, 95%CI:0**.6**8–10.98) and hospital duration (OR = 1.03, 95%CI: 1.01–1.06) were significant risk factors for NI. Multivariate also showed the same three significant risk factors, including weight (OR = 1.1, 95%CI: 1.04–1.18), peritoneal dialysis (OR = 2.9, 95%CI: 1.12–5.87), and hospital duration (OR = 1.03, 95%CI: 1.01–1.07). For surgical site wound infection, we did not find significant risk factors which might be attributed to the limited sample size.

**Table 4:** Univariate analysis and multivariate analysis of risk factors for Nosocomial infections during ECMO.

**Factors**	**Univariate analysis**			**Multivariate analysis**		
	**OR**	**95%CI**	***P*–value**	**OR**	**95%CI**	***P*–value**
Age	0.85	0.72–1.16	0.053	0.88	0.73–1.08	0.076
Weight	0.38	0.07–1.84	0.241	0.39	0.10–1.13	0.082
Peritoneal dialysis	2.72	1.15–6.43	0.022^*^	2.69	1.08–5.73	0.031^*^
ECMO support mode	1.84	1.43–2.45	0.001^*^	1.71	0.86–2.39	0.554
ECMO duration (d)	2.23	1.08–6.38	0.036^*^	2.46	1.10–5.51	0.029^*^
IMV duration (d)	1.49	0.82–1.86	0.452	1.47	0.98–1.64	0.063
ICU duration (d)	1.33	1.04–1.65	0.024^*^	1.35	1.05–1.59	0.017^*^
Hospital duration (d)	1.08	0.91–1.43	0.217	1.10	0.92–1.54	0.768
**Respiratory tract infection**						
Age	0.95	0.85–1.05	0.347	1	0.84–1.14	0.982
Weight	0.45	0.21–1.06	0.361	0.49	0.38–0.55	0.751
Peritoneal dialysis	0.89	0.25–2.84	0.849	2.49	0.5–12.5	0.257
ECMO support mode	1.68	1.41–4.14	0.026^*^	2.45	0.28–65.33	0.355
ECMO duration (d)	1.32	1.16–1.56	0.001^*^	1.38	1.17–1.73	0.001^*^
IMV duration (d)	1.04	1–1.08	0.032^*^	0.98	0.91–1.06	0.602
ICU duration (d)	1.02	0.99–1.06	0.125	0.97	0.87–1.06	0.499
Hospital duration (d)	0.02	1–1.05	0.051	1.05	1–1.14	0.178
**Blood stream infection**						
Age	1.07	0.98–1.17	0.112	0.97	0.84–1.08	0.575
Weight	1.05	1.01–1.09	0.017^*^	1.1	1.04–1.18	0.001^*^
Peritoneal dialysis	1.33	0.68–10.98	0.048^*^	2.9	1.12–5.87	0.005^*^
ECMO support mode	1.98	1.55–3.21	0.993	0	0.35–4.55	0.993
ECMO duration (d)	0.97	0.8–1.13	0.701	0.83	0.63–1.04	0.133
IMV duration (d)	1.04	0.95–1.12	0.406	1.17	0.98–1.42	0.087
ICU duration (d)	1.03	0.97–1.1	0.356	0.93	0.8–1.06	0.287
Hospital duration (d)	1.03	1.01–1.06	0.016^*^	1.03	1.01–1.07	0.027^*^
**Surgical site wound infection**						
Age	0.91	0.74–1.06	0.271	0.99	0.74–1.16	0.603
Weight	0.97	0.89–1.06	0.497	0.51	0.87–1.69	0.511
Peritoneal dialysis	0.94	0.11–9.1	0.952	5.55	0.06–15.83	0.561
ECMO support mode	2.52	1.98–4.42	0.995	0.85	0.25–1.85	0.997
ECMO duration (d)	0.98	0.85–1.14	0.789	1	0.81–1.24	0.985
IMV duration (d)	0.98	0.93–1.02	0.422	0.77	0.39–1.04	0.314
ICU duration (d)	0.99	0.95–1.03	0.562	1.32	0.96–2.74	0.316
Hospital duration (d)	0.99	0.96–1.01	0.271	0.97	0.87–1	0.301

* *P–value < 0.05*.

## Discussion

In this study, we retrospectively analyzed the incidence and risk factors of NI among a large number of pediatric patients receiving ECMO for refractory respiratory or cardiac failure. Our results showed that 28% of patients developed at least one episode of NI during ECMO, which was consistent with the previous results of 20–45% for pediatric and adults patients (19–21, 23). The incidence of NI in this study was 47.6 cases per 1,000 ECMO days, which was in accordance with the previously reported values of between 10 and 116.2 per 1,000 ECMO days (19–21, 23, 24). The prolonged ECMO duration, ICU duration, and peritoneal dialysis were independent factors associated with the development of NI during ECMO.

To the best of our knowledge, this was the first study to investigate the epidemiology and risk factors of NIs in pediatric population who underwent ECMO for respiratory and/or circulatory failure in China. In the present study, we found that gram-negative pathogens were the dominant causative pathogens of respiratory tract infections and BIS during ECMO cannulation, which accounted for 92.6% of nosocomial pathogens. This result was higher than the value of 78% reported by Hsu et al. (25) in adult patients. However, previous studies showed that gram-positive *cocci*, especially coagulase-negative *staphylococci*, were the most frequent pathogen of bloodstream infection during ECMO (19, 21). Hsu et al. stated that the possible reason for the difference between theirs and previous studies was the early administration of glycopeptides and selection of gram-negative pathogens (25).

A variety of studies have assessed the risk factors for developing NI in patients who underwent ECMO. These factors included prolonged duration of ECMO (20–22), venoarterial ECMO (14) and central cannulation (26). In this study, we found that the prolonged duration of ECMO support was an independent risk factor for NI in patients who underwent ECMO. Our result was confirmed by the previous studies (14, 25). Bizzarro et al. conducted a retrospective study on 20,741 patients and found that longer ECMO support was significantly associated with the NI irrespective of age. Patients who underwent ECMO for less than 1 week had a 6.1% risk of NI, as compared with 15.7% of those receiving ECMO for between 8 and 14 days, and 30.3% for those receiving ECMO for more than 2 weeks (*P* **<** 0.001) (14). For adult patients, the risk of NI was 12.8% in those with ECMO for < a week, compared to 51.6% in those with ECMO for more than 2 weeks (*P* **<** 0.001) (14). Hsu et al. also found that the NI rate increased three-fold when the duration of ECMO exceed more than 10 days (5.7 vs. 19.2%) (25). The ICU duration was also found to be an independent risk factor for NI in the multivariate analysis, however, its effect was not as strong as the ECMO duration (OR = 1.35 vs. 2.46). Furthermore, concerning respiratory tract infection, ECMO support mode and duration along with IMV duration need attention. In BSI, weight, peritoneal dialysis, and hospital duration might be risk factors for NI. These findings were partly consistent with previous studies (24, 25).

The clinical diagnosis of NI in ECMO patients is challenging because observable symptoms/signs of the patients may not be present. Fever is often non-apparent since body temperature is controlled by an ECMO heat exchanger and patients invariably have signs of the systemic inflammatory response, possibly triggered by ECMO itself (21, 27). For this reason, we only analyzed patients with microbiologically confirmed infections. Concerning the causative microorganisms, previous research showed that *Staphylococcus aureus* and other *Staphylococcus* species (38%) were the most common pathogens causing BSI, followed by *Escherichia coli* (24%), in critically ill patients without ECMO support (28, 29). However, the balance was shifting from multi-resistant *staphylococci* to highly resistant classes of *A. baumannii* and *P. aeruginosa*. In our study, *A. baumannii* was the most common pathogen in both blood and the respiratory tract, which would be a common pathogen in the ICU environment. We also found 3 cases of developed fungal infections, which accounted for 5.6% of nosocomial pathogens. Similarly, Giacomo et al. (30) reported 17% of the fungal infection during ECMO support. The shift from G-positive to G-negative bacteria can be possibly explained by the increased antimicrobial exposure, intestinal microbiota selection during the hospital stay (31), and the gut mucosal barrier impairment.

There were several potential limitations in this study. First, this study was a multicenter retrospective cohort study, and as such, it can only suggest associations of risk factors without inferring reasons for NI (32). Second, although cases in this study were enrolled, the clinical information of risk factors was collected retrospectively, which might result in a selection bias. Third, the retrospective nature of this cohort study is limited to medical patients only, which precludes the extrapolation of the results to the general population of patients with medical ECMO. Fourth, due to the lack of sufficient data about the patient outcome, we are not able to investigate the prognostic factors of Nis in these patients.

In conclusion, this study investigated the epidemiology and risk factors of NI in pediatric patients who underwent ECMO for respiratory and/or circulatory failure. Risk factors for NI during ECMO included a longer duration of ECMO and ICU and peritoneal dialysis. Given that all the available data are obtained retrospectively, a prospective study focusing on this topic is needed to verify our findings.

## Data Availability Statement

The original contributions presented in the study are included in the article/Supplementary Material, further inquiries can be directed to the corresponding author.

## Ethics Statement

The studies involving human participants were reviewed and approved by the Institutional Ethical Committee of the Seventh Medical Center of PLA General Hospital in China. Written informed consent to participate in this study was provided by the participants or their legal guardian/next of kin.

## Author Contributions

ZW and YX attributed to the study deign. CW, SL, FW, and JY contributed to data collection. CW, SL, WY, and XG did the data analyses. CW, SL, and YX wrote the manuscript. All authors read and approved the final manuscript.

## Conflict of Interest

The authors declare that the research was conducted in the absence of any commercial or financial relationships that could be construed as a potential conflict of interest.

## Publisher's Note

All claims expressed in this article are solely those of the authors and do not necessarily represent those of their affiliated organizations, or those of the publisher, the editors and the reviewers. Any product that may be evaluated in this article, or claim that may be made by its manufacturer, is not guaranteed or endorsed by the publisher.
